# Development and validation of a new score for predicting functional outcome of neurocritically ill patients: The INCNS score

**DOI:** 10.1111/cns.13134

**Published:** 2019-04-10

**Authors:** Qiong Gao, Fang Yuan, Xi‐Ai Yang, Ji‐Wen Zhu, Lu Song, Li‐Jie Bi, Ze‐Yu Jiao, Xiao‐Gang Kang, Fang Yang, Wen Jiang

**Affiliations:** ^1^ Department of Neurology Xijing Hospital, Fourth Military Medical University Xi’an China

**Keywords:** APACHE II, INCNS, neurocritical illness, prognostic score, SAPS II

## Abstract

**Aims:**

To develop and validate a novel score for prediction of 3‐month functional outcome in neurocritically ill patients.

**Methods:**

The development of the novel score was based on two widely used scores for general critical illnesses (Acute Physiology and Chronic Health Evaluation II, APACHE II; Simplified Acute Physiology Score II, SAPS II) and consideration of the characteristics of neurocritical illness. Data from consecutive patients admitted to neurological ICU (N‐ICU) between January 2013 and June 2016 were used for the validation. The modified Rankin Scale (mRS) was used to evaluate 3‐month functional outcomes. APACHE II scores, SAPS II scores, and our novel scores at 24 hours and 72 hours in N‐ICU were obtained. We compared the prognostic performance of our score with APACHE II and SAPS II.

**Results:**

We developed a 44‐point scoring system named the INCNS score, and it includes 19 items which were categorized into five parts: inflammation (I), nutrition (N), consciousness (C), neurological function (N), and systemic function (S). We validated the INCNS score with a cohort of 941 N‐ICU patients. The 72‐hours INCNS score achieved an area under the receiver operating characteristic curve (AUC) of 0.828 (95% CI: 0.802‐0.854), and the 24‐hours INCNS score achieved an AUC of 0.788 (95% CI: 0.759‐0.817). The INCNS score exhibited significantly better discriminative and prognostic performance than APACHE II and SAPS II at both 24 hours and 72 hours in N‐ICU.

**Conclusion:**

We developed an INCNS score with superior predictive power for functional outcome of neurocritically ill patients.

## 1 INTRODUCTION

Neurocritical illness, defined as life‐threatening neurological and neurosurgical illness, is a catastrophic event with high mortality and prolonged functional dependence.^1^, ^2^, ^3^ An accurate and specialized prognostic score for neurocritical illness will provide clinicians a useful overview to summarize clinical findings, help stratify patients in critical care researches, and facilitate more effective communication with patients and their families about goals of care, choices of treatments, and plans for transitions.

To date, there are several prognostic scores for patients in general intensive care unit (ICU), such as the Acute Physiology and Chronic Health Evaluation II (APACHE II)^4^ and Simplified Acute Physiology Score II (SAPS II).^5^ Meanwhile, some scales were also developed for specific critical illnesses, such as the Ventilator‐Associated Pneumonia PIRO Score,^6^ Obstetric Early Warning Score,^7^ Oncological Pediatric Risk of Mortality Score (O‐PRISM),^8^ the Edinburgh Cardiac Surgery Score,^9^ Initiation of Renal Replacement Therapy (IRRIV) Score^10^ and so on. However, so far there is no prognostic score for patients with neurocritical illness who have characteristics distinct from that of patients with other critical illnesses. And the lack of assessment of neurological deficits in those risk adjustment models biases the severity evaluation and outcome prediction in patients with neurocritical illnesses.

Glasgow coma scale (GCS)^11^ and the Full Outline of UnResponsiveness (FOUR) score^12^ are common methods for bedside assessment of impairment of conscious level,^13^ and are widely used in neurological intensive care unit (N‐ICU). Although these two scales are strongly associated with severity and outcome which are assessed by other indices,^14^, ^15^, ^16^ the assessment of consciousness per se is unable to make comprehensive predictive statements.

Given the paucity of prognostic scores in neurocritical illness, we aimed to develop a novel score for prediction of 3‐month functional outcome in neurocritically ill patients, and compare it with APACHE II and SAPS II in a cohort of neurocritically ill patients.

## 2 METHODS

### 2.1 Hypothesis generation

**Figure 1:**
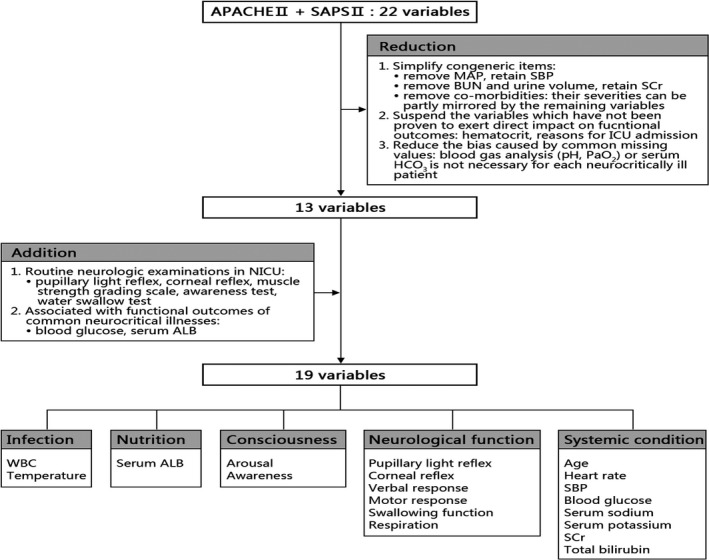
Flow chart for the variable generation of the INCNS score. *ALB* albumin, *APACHE II* Acute Physiology and Chronic Health Evaluation II, *BUN* blood urea nitrogen, *MAP* mean artery pressure, *N‐ICU* neurological intensive care unit, *SAPS II* Simplified Acute Physiology Score II, *SBP* systolic blood pressure, *SCr* serum creatinine, *WBC* white blood cell

We hypothesized that a specialized score for neurocritical illness would be the combination of systemic condition evaluation and neurological function assessment. Since the variables in APACHE II and SAPS II were derived from expert consensus and large cohorts of general critical illness^4^, ^5^ and have been validated all over the world,^17^, ^18^, ^19^, ^20^, ^21^ we believed that the integration and modification of these two scores would provide a reliable and reasonable basis for evaluating systemic condition accurately. Measurements concerning different aspects of neurological deficits and being routinely used in N‐ICU were also considered. The process of the generation of the novel score named INCNS is depicted in Figure 1.

### 2.2 Variable selection

On the basis of 22 different variables in APACHE II and SAPS II, reductions and additions were made in order to form a prognostic score for neurocritical illnesses with good accuracy, availability, and ease of use. The reduction procedure followed three principles: (a) simplify congeneric items (systolic blood pressure was retained, and mean arterial pressure was removed; serum creatinine was retained, and blood urea nitrogen and urine volume were removed; co‐morbidities were removed because their severities could be partly mirrored by the remaining variables); (b) suspend the variables which have not been fully proven to exert direct impacts on functional outcomes of neurocritical illnesses (hematocrit was removed; reasons for ICU admission were also removed, because some patients who don’t need emergency surgery might have much poorer outcomes than those who need, such as the patients with acute intoxication or cardiac arrest for instance); (c) reduce the bias caused by common missing values: blood gas analysis (pH, PaO2) or serum HCO_3_ is not necessary for each neurocritically ill patient. The addition procedure followed two principles: (a) incorporate some routine neurologic examinations in N‐ICU (papillary light reflex, corneal reflex, muscle strength test, consiousness test, and water swallow test); (b) include biomarkers which are associated with functional outcomes of common neurocritical illnesses (blood glucose and serum albumin). Previous studies proved that abnormal blood glucose and serum albumin levels were independently related to the mortality and morbidity in some common neurologic diseases, such as stroke^22^, ^23^, ^24^, ^25^, ^26^, ^27^, ^28^, ^29^ and status epilepticus.^30^, ^31^ After the above reduction and addition procedures, 19 variables were finally determined. In order to make those variables easier to understand and remember, we grouped them into five categories: inflammation, nutrition, consciousness, neurological function and systemic condition (INCNS for short).

### 2.3 Definition of the variables

White blood cell, serum albumin, random blood glucose, serum sodium, serum potassium, serum creatinine, and total bilirubin are routine labs. Temperature, respiration, heart rate, and systolic blood pressure are vital signs. Consciousness is evaluated from two aspects: (a) arousal, assessed by eye opening test (spontaneous, to verbal command, to pain, and no response); (b) awareness, assessed by behavioral tests (correct or confused behavioral responses to question or command) and tests for non‐reflex movements (visual pursuit or non‐contingent behaviors). The assessment of arousal status is derived from GCS which is included in APACHE II and SAPS II. The behavioral tests for awareness are routine bedside examinations in N‐ICU. The examiner may ask a question about the patient’s name or command the patient to move eyeballs and/or hands, if appropriate. A clear and accurate answer was considered correct, and a clearly intentional communicative response, such as head nods/shakes and thumbs up, was considered as confused. For the patients who are unable to communicate, tests for non‐reflex movements were performed. Non‐reflex movements include visual pursuit, orientation to pain or non‐contingent behavior (eg smiling to the presence of a family member and not to others).^32^ Neurologic function is evaluated from six aspects: (a) pupillary light reflex; (b) corneal reflex; (c) verbal response; (d) motor response; (e) swallowing function; (f) respiration. The pupillary light reflex and corneal reflex are brainstem reflexes that test mesencephalon and pons function. The assessments of verbal and motor function are derived from GCS which is included in APACHE II and SAPS II. For the intubated patients, their verbal function is scored as no verbal response. The muscle strength test is also used to assess the motor function. Either the muscle strength test or motor response to painful stimulus is performed in each patient. Patients who are unable to cooperate with muscle strength test choose the motor response to painful stimulus test. To be noted, unlike GCS score that records the best motor response from any limb, the worst motor response from any limb is recorded in our score. Swallowing function is assessed by the 30 mL water swallow test. In APACHE II the respiratory function is measured only by the respiratory rate no matter the patient is ventilated or non‐ventilated. However, the respiratory function of the ventilated patients usually is much poorer than those who don’t need mechanical ventilation. Therefore, we classified the respiratory function into four degrees: (a) not intubated, respiratory rate of 12 to 24; (b) not intubated, respiratory rate ≤ 11 or ≥25; (c) breathes above ventilator rate; (d) breathes at ventilator rate or apnea.

### 2.4 Weight assignment

In order to make this score easy to calculate and use, the weighing system was based on a simple scale of 0 to 3 (like APACHE II) instead of a sophisticated scale of irregular numbers (like SPAS II) generated by statistic calculation. 0 denotes normal, and a higher score means a more serious condition. Considering that brainstem function tests play a pivotal role in outcome prediction, we assigned higher scores to the slow and absent responses of papillary light reflex and corneal reflex.

### 2.5 Population

This analysis was based on a prospective database of all the patients admitted to N‐ICU in Xijing Hospital, a tertiary academic medical care institution with 3200 beds in Xi’an, China. Consecutive patients who have stayed in N‐ICU for longer than 72 hours between January 2013 and June 2016 were used for the validation of the INCNS score. The ethics committee of the Xijing hospital reviewed and approved the study. All the procedures were carried out in agreement with Chinese laws and the Helsinki declaration relative to patients’ rights.

### 2.6 Data collection

The data concerning all the variables included in APACHE II, SAPS II, and INCNS score were reviewed. The most deranged reading during each patient’s initial 24 hours and 72 hours in N‐ICU were recorded and used in the validation analysis. All the neurologic examinations involved in these three scores are daily routines in N‐ICU, and were performed by the neurologist who was in charge of each patient.

### 2.7 Definition of outcomes

The modified Rankin Scale (mRS), a seven‐point scale ranging from 0 (no symptoms) to 6 (death), was used to evaluate the functional outcome 3 months after the discharge from N‐ICU, by a trained research assistant who was blinded to clinical data. A mRS score < 3 was considered favorable (independence), and a mRS score ≥ 3 was considered unfavorable (dependence or death).

### 2.8 Statistical analysis

Continuous variables were expressed as mean ± SD or median (interquartile range, IQR), and categorical variables were expressed as percentages. Continuous variables were analyzed using Student’s t test for normally distributed variables, and Mann ‐ Whitney *U* test for non‐normally distributed variables. Categorical variables were analyzed using *χ* ^2^ test analysis, and Fisher’s exact tests, when appropriate. All the above tests were two‐sided, and a *P* value < 0.05 was considered statistically significant.

The receiver operating characteristics curve (ROC) analysis was used to determine the predictive power of APACHE II, SAPS II, GCS, FOUR and INCNS score. Sensitivity, specificity, positive (PPV), negative predictive value (NPV), number of correctly classified (CC) patients and the maximum accuracy determined by cut‐off values (Youden Index) were calculated for INCNS, APACHE II, and SAPS II. Mc Nemar’s test was used to compare sensitivity, specificity and CC.^33^ A modification of Wald tests was used to examine the significance of difference of PPV and NPV.^34^ A *P* value <0.0167 for multiple comparison tests on performances was considered statistically significant.^35^ The probability values of predicting poor prognosis of INCNS score were derived from the logistic regression model. Statistical analyses were performed using SPSS 22.0 and Matlab 2012a.

## 3 RESULTS

The INCNS score was presented in Table 1.

**Table 1:** Scoresheet of the INCNS score

	Variable	Points			
		0	1	**2**	**3**
Inflammation	WBC (10^9^/L)	4 ~ 10	2.9 ~ 3.9, 10.1 ~ 25.0	≤2.8, ≥25.1	—
	Temperature (axillary, °C)	36 ~ 38.4	≤35.9, 38.5 ~ 40	≥40.1	—
Nutrition	Albumin (g/L)	≥35	25 ~ 34.9	≤24.9	—
Consciousness	Arousal	Spontaneous eye opening	Eye opening to verbal command	Eye opening to pain	None
	Awareness	Correct response to question or command^a^	confused response to question or command^a^	Non‐reflex movements^b^	None
Neurologic function	Pupillary light reflex	Bilateral sensitive	—	Unilateral slow/absent	Bilateral slow/absent
	Corneal reflex	Bilateral sensitive	—	Unilateral slow/absent	Bilateral slow/absent
	Verbal response	Accurate speech	Confused/inappropriate speech	Incomprehensible speech/none	—
	Motor response^c^	Unilateral/bilateral muscle strength scores ≥ 4	Unilateral/bilateral muscle strength scores of 2‐3	Unilateral muscle strength scores ≤ 1	Bilateral muscle strength scores ≤ 1
		Obeying to command	Localizing to/withdrawal from pain	Flexing/extending to pain	None
	Swallowing function	Water swallow test I‐II	Water swallow test III‐IV/unable to assess	—	—
	Respiration	Not intubated, 12 ~ 24	Not intubated, ≤11/≥25	Breathes above ventilator rate	Breathes at ventilator rate/ apnea
Systemic condition	Age (y)	≤44	45 ~ 64	65 ~ 74	≥75
	Heart rate	60 ~ 100	40 ~ 59, 101 ~ 149	≤39, ≥ 150	—
	SBP (mm Hg)	90 ~ 140	70 ~ 89, 141 ~ 199	≤69, ≥ 200	—
	Blood glucose (mmol/L)	3.9 ~ 11.1	2.2 ~ 3.8, 11.2 ~ 19.3	≤2.1, ≥ 19.4	—
	Serum sodium (mmol/L)	130 ~ 150	120 ~ 129, 151 ~ 159	≤119, ≥ 160	—
	Serum potassium (mmol/L)	3.5 ~ 5.5	2.5 ~ 3.4, 5.6 ~ 6.9	≤2.4, ≥ 7.0	—
	Serum creatinine (μmol/L)	44 ~ 132	≤43, 133 ~ 171	≥172	—
	Total bilirubin (μmol/L)	≤34.1	34.2 ~ 102.5	≥102.6	—

SBP, systolic blood pressure; WBC, white blood cell.

a The examiner may ask a question about the patient’s name or command the patient to move eyeballs and/or hands, if appropriate.

b Include evidence of visual pursuit or non‐contingent behaviors.

c Either the muscle strength test or motor response to painful stimulus is performed in each patient.

### 3.1 Validation cohort

From January 2013 and June 2016, a total of 1037 patients were admitted to our N‐ICU and had stayed for longer than 3 days. Ninety‐six patients were lost to follow‐up, and 941 patients were enrolled for the analysis. The median age for the cohort was 52 (IQR, 37‐64) years, and 59.9% were male. The median hospital length of stay was 15 (IQR 9‐23) days, and the median length of N‐ICU stay was 10 (IQR 6‐16) days. At 3 months post N‐ICU discharge, 384 (40.8%) patients attained favorable outcomes (mRS 0‐2), while 557 (59.2%) patients had unfavorable outcomes (mRS 3‐6), among whom 232 (24.7%) died. Baseline characteristics of patients and the worst values of all variables during initial 24‐hours and 72‐hours in N‐ICU were presented in Tables S1 and S2 respectively. The etiology distribution in this cohort was provided in Table S3.

### 3.2 Predictive performance of INCNS score

**Figure 2:**
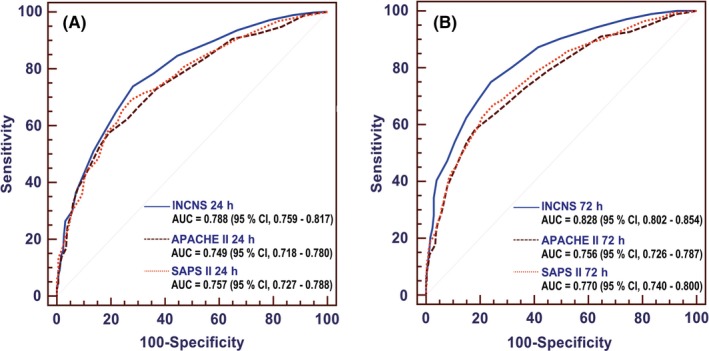
Comparisons of area under the receiver operating characteristic curve (AUC) for INCNS, Acute Physiology and Chronic Health Evaluation II (APACHE II) and Simplified Acute Physiology Score II (SAPS II) to discriminate the 3‐mo functional outcome in neurocritically ill patients. A, ROC at 24 h: the *P* value for the comparison of AUC between INCNS and APACHE II is 0.0011, between INCNS and SAPS II is 0.0117. B, ROC at 72 h: the *P* value for the comparison of AUC between INCNS and APACHE II is <0.0001, between INCNS and SAPS II is also <0.0001. Level of significance corrected for multiple testing *P* < 0.0167

**Figure 3:**
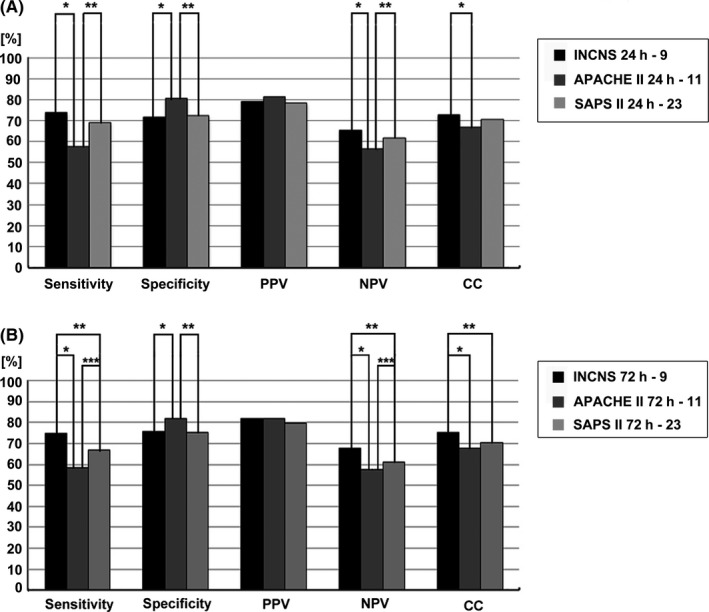
Comparisons of sensitivity, specificity, positive predictive value (PPV), negative predictive value (NPV) and number of correctly classified (CC) patients between INCNS, Acute Physiology and Chronic Health Evaluation II (APACHE II) and Simplified Acute Physiology Score II (SAPS II) to identify the predictive performance of 3‐mo functional outcome in neurocritically ill patients. Sensitivity: *P = 0.000000000000000036, **P = 0.0000043, ***P = 0.000022; specificity: *P = 0.0081, **P = 0.0041; NPV: *P = 0.00000000013, **P = 0.000020, ***P = 0.013; CC: *P = 0.00028, **P = 0.015. Level of significance corrected for multiple testing *P* < 0.0167

**Figure 4:**
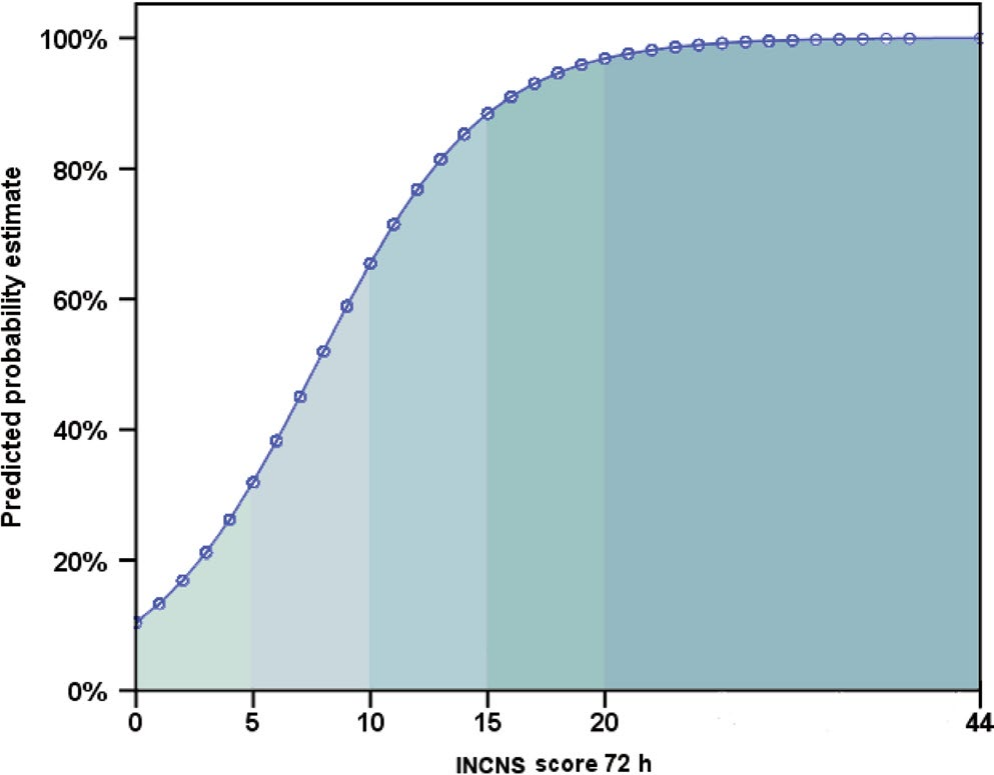
Points and their corresponding predicted estimates of the 3‐mo unfavorable outcome of neurological intensive care unit (N‐ICU) patients based on the 72‐h INCNS score

The 24‐hours INCNS scoring system yielded an area under the receiver operating characteristic curve (AUC) of 0.788 (95% CI, 0.759‐0.817), and the 72‐hours INCNS score yielded an AUC of 0.828 (95% CI, 0.802‐0.854) (Figure 2, Table S4). The cut‐off value determined by the maximum sum of sensitivity and specificity was nine for both 24‐hours and 72‐hours INCNS scores. Using the cutoff score, the 72‐hours INCNS score predicted 3‐month unfavorable outcomes with a predictive accuracy of 75.5% (sensitivity: 75.0%, specificity: 76.0%, PPV: 82.0%, NPV: 67.7%, CC: 75.5%), and the 24‐hours INCNS score had a predictive accuracy of 72.9% (sensitivity: 73.8%, specificity: 71.9%, PPV: 79.2%, NPV: 65.4%, CC: 73%) (Figure 3, Table S4). Figure 4 showed that the probability of an unfavorable outcome of neurocritically ill patients increased as the 72‐hours INCNS score rose.

### 3.3 Comparison of INCNS, APACHE II and SAPS II score

Overall performances of INCNS, APACHE II and SAPS II were presented in Figure 2, Figure 3 and Table S4. According to the results of ROC analyses, the 72‐hours INCNS scoring system had the greatest discriminative power and highest predictive accuracy, followed by the 24‐hours INCNS, 72‐hours SAPS II, 24‐hours SAPS II, 72‐hours APACHE II and 24‐hours APACHE II scoring systems. At both 24 hours and 72 hours in N‐ICU, the INCNS score had significantly greater discriminative power than APACHE II (24 hours: *P* = 0.0011; 72 hours: *P* < 0.0001) and SAPS II (24 hours: *P* = 0.0117; 72 hours: *P* < 0.0001). The sensitivity, NPV, and CC of the INCNS score were also significantly higher than those of APACHE II and SAPS II at both 24 hours and 72 hours (Figure 3, Table S4). Moreover, the INCNS score showed much better discriminative performance compared with GCS (24 hours: *P* < 0.0001; 72 hours: *P* < 0.0001) and FOUR (24 hours: *P* < 0.0001; 72 hours: *P* < 0.0001) at both 24 hours and 72 hours as well (Figure S1).

## 4 DISCUSSION

Based on two widely used scores for critical illness (APACHE II and SAPS II) and consideration of the characteristics of neurocritical illness, we developed a novel clinically useful yet statistically accurate and valid score for predicting functional outcome in neurocritically ill patients. We named it INCNS, which is an acronym for inflammation, nutrition, consciousness, neurological function and systemic function. Data from 941 neurocritically ill patients validated that the INCNS score had significantly greater predictive ability than APACHE II and SAPS II.

In this study, multivariate logistic regression was not used to identify predictor variables in the INCNS score, because the limited sample size and population diversity would bias the conclusions of the regression model. The major measurements of the INCNS score were derived from APACHE II and SAPS II, which have been validated in abundant cohorts worldwide.^17^, ^18^, ^19^, ^20^, ^21^ This provided a robust support for the INCNS score to be used reliably in different regions where different distributions of neurological illness, age, race may be encountered.

The verbal and motor response tests in INCNS were also derived from GCS, however, we made several modifications to it for the purpose of evaluating verbal and motor ability rather than merely consciousness level. For intubated patients, we believed that the use of intubation would inevitably affect their language, therefore they were scored as no response in the verbal response test in INCNS score. Besides, since the motor response to painful stimulus test in GCS was designed for comatose patients, we combined muscle strength test with the motor response to painful stimulus test to evaluate the motor function. Only one of these two tests was performed for each patient according to his ability, and the examiner may choose the appropriate one. Moreover, the scoring method in the motor response test was also modified. The worst motor response from any limb rather than the best one is recorded in our score, because the purpose of the INCNS score is to predict 3‐month functional outcome, and the disability of any limb will definitely impair motor skills.

Arousal and awareness are essential parts of consciousness. Arousal is the psychological and physiological state of being awoken, and awareness is the ability to perceive or to be cognizant of the self‐existence and events. In INCNS score, we used the eye opening test, behavioral tests and tests for non‐reflex movements to evaluate the levels of arousal and awareness. The INCNS score also includes the assessments of brainstem reflexes and swallowing function. The pupillary light reflex and corneal reflex are routine neurologic examinations in N‐ICU and convenient approaches for assessing the function of brainstem, which plays a pivotal role in maintaining many basic functions, such as consciousness, breathing, heart rate, and sleeping. As a common result of a variety of neurological diseases (cerebral infarction, intracerebral hemorrhage, traumatic brain injury, myasthenia gravis, dementia, etc),^36^ dysphagia is associated with some unfavorable outcomes, including pneumonia, reintubation, in‐hospital mortality and prolonged hospital length of stay.^36^, ^37^, ^38^, ^39^ Moreover, the difficulty in swallowing will also affect the quality of life in long term. Therefore, the water swallow test was adopted to assess the swallowing function. All the neurologic examinations used in INCNS score are common and routine tests, and have been proven to possess excellent interrater reliability.^40^, ^41^, ^42^

APACHE II and SAPS II were established based on the worst values of the measurements during the first 24 hours in general ICU.^4^, ^5^ However, some studies suggested that a severity score (APACHE II or the mortality probability models) that performed at 72 hours in ICU worked much better than the one performed on admission, 24 hours or 48 hours.^43^, ^44^ In this study, predictive performances of both 24‐hours and 72‐hours INCNS scores were examined in 941 N‐ICU patients, and they were compared with the performances of APACHE II and SAPS II. Results from our studies showed that INCNS score had significantly stronger predictive power in discriminative power, sensitivity, NPV, and CC than APACHE II and SAPS II at both 24 hours and 72 hours in N‐ICU, however, APACHE II score had a greater specificity with respect to the other two scores. Specificity quantified the proportion of patients with actual favorable outcome that were correctly identified as such. Therefore, compared to APACHE II score, INCNS score and SAPS II score had a higher rate of false positives, which indicated that more people with favorable outcome were predicted wrongly as the ones with unfavorable outcome. Our study also confirmed that the INCNS score performed at 72 hours achieved better accuracy than the one performed at 24 hours, which might be partially because the progressive worsening usually extends beyond 24 hours after the admission to ICU.

Several weaknesses of the current study should be noted. First, the selection of predictor variables was based on previous studies, clinical experience, and usability considerations, instead of statistical calculation. To some extent, it may be arbitrary. However, the drawbacks of a limited derivation cohort (eg in number and diversity of population) could be avoided in this way. Second, weight assignment was based on a simple scale of 0 to 3 instead of numbers generated by statistic analysis. Although it made INCNS easier to calculate, it may compromise the overall accuracy. Third, with 19 items, the INCNS score is a little unwieldy. However, INCNS score already has less total scores and items than APACHE II and SAPS II, and all the items in INCNS score are routine labs and neurologic examinations in N‐ICU. Thus, rating the INCNS score won’t bring extra work. Moreover, patients receiving sedative agents or neuromuscular function blockers were not excluded. The validation results in those patients may be biased by the overestimation of their consciousness impairment. However, this is inevitable in the real world. Future studies are required to further examine the performance of INCNS score in more diverse populations.

## 5 CONCLUSIONS

We established a novel risk prediction score, named INCNS, to predict the 3‐month functional outcome of neurocritically ill patients. The INCNS score integrates measurements from five aspects: inflammation (I), nutrition (N), consciousness (C), neurologic function (N) and systemic condition (S). Data from a cohort of 941 neurocritically ill patients showed that INCNS score had significantly stronger predictive power than APACHE II and SAPS II at both 24 hours and 72 hours in N‐ICU.

## CONFLICT OF INTEREST

The authors declare no conflict of interest.

## References

[1] Broessner G , Helbok R , Lackner P , et al. Survival and long‐term functional outcome in 1,155 consecutive neurocritical care patients. Crit Care Med. 2007;35(9):2025‐2030. 17855816 10.1097/01.ccm.0000281449.07719.2b

[2] Kiphuth IC , Schellinger PD , Köhrmann M , et al. Predictors for good functional outcome after neurocritical care. Crit Care. 2010;14(4):R136. 20646313 10.1186/cc9192 PMC2945110

[3] Huang M , Wang J , Ni X , Chen G , Kong L . Neurocritical care in China: past, present, and future. World neurosurg. 2016;95:502‐506. 27373935 10.1016/j.wneu.2016.06.102

[4] Knaus WA , Draper EA , Wagner DP , Zimmerman JE . APACHE II: a severity of disease classification system. Crit Care Med. 1985;13(10):818‐829. 3928249

[5] Le Gall JR , Lemeshow S , Saulnier F . A new simplified acute physiology score (SAPS II) based on a European/North American multicenter study. JAMA. 1993;270(24):2957‐2963. 8254858 10.1001/jama.270.24.2957

[6] Lisboa T , Diaz E , Sa‐Borges M , et al. The ventilator‐associated pneumonia PIRO score: a tool for predicting ICU mortality and health‐care resources use in ventilator‐associated pneumonia. Chest. 2008;134(6):1208‐1216. 18779186 10.1378/chest.08-1106

[7] Carle C , Alexander P , Columb M , Johal J . Design and internal validation of an obstetric early warning score: secondary analysis of the intensive care national audit and research centre case mix programme database. Anaesthesia. 2013;68(4):354‐367. 23488833 10.1111/anae.12180

[8] Schneider DT , Lemburg P , Sprock I , Heying R , Göbel U , Nürnberger W . Introduction of the oncological pediatric risk of mortality score (O‐PRISM) for ICU support following stem cell transplantation in children. Bone Marrow Transplant. 2000;25(10):1079‐1086. 10828869 10.1038/sj.bmt.1702403

[9] Thompson M , Elton R , Sturgeon K , et al. The Edinburgh cardiac surgery score survival prediction in the long‐stay ICU cardiac surgical patient. Eur J Cardiothorac Surg. 1995;9(8):419‐425. 7495585 10.1016/s1010-7940(05)80076-4

[10] Zaragoza JJ , Villa G , Garzotto F , et al. Initiation of renal replacement therapy in the intensive care unit in Vicenza (IRRIV) score. Blood purif. 2015;39(1–3):246‐257. 25925505 10.1159/000381009

[11] Teasdale G , Jennett B . Assessment of coma and impaired consciousness. A practical scale. Lancet. 1974;2(7872):81‐84. 4136544 10.1016/s0140-6736(74)91639-0

[12] Wijdicks EF , Bamlet WR , Maramattom BV , Manno EM , McClelland RL . Validation of a new coma scale: the FOUR score. Ann Neurol. 2005;58(4):585‐593. 16178024 10.1002/ana.20611

[13] Bordini AL , Luiz TF , Fernandes M , Arruda WO , Teive HA . Coma scales: a historical review. Arq Neuropsiquiatr. 2010;68(6):930‐937. 21243255 10.1590/s0004-282x2010000600019

[14] Collaborators M , Perel P , Arango M , et al. Predicting outcome after traumatic brain injury: practical prognostic models based on large cohort of international patients. BMJ. 2008;336(7641):425‐429. 18270239 10.1136/bmj.39461.643438.25 PMC2249681

[15] Okasha AS , Fayed AM , Saleh AS . The FOUR score predicts mortality, endotracheal intubation and ICU length of stay after traumatic brain injury. Neurocrit Care. 2014;21(3):496‐504. 24865273 10.1007/s12028-014-9995-6

[16] McNett M , Amato S , Gianakis A , et al. The FOUR score and GCS as predictors of outcome after traumatic brain injury. Neurocrit Care. 2014;21(1):52‐57. 24408147 10.1007/s12028-013-9947-6

[17] Beck DH , Smith GB , Pappachan JV , Millar B . External validation of the SAPS II, APACHE II and APACHE III prognostic models in South England: a multicentre study. Intensive Care Med. 2003;29(2):249‐256. 12536271 10.1007/s00134-002-1607-9

[18] Arabi Y , Haddad S , Goraj R , Al‐Shimemeri A , Al‐Malik S . Assessment of performance of four mortality prediction systems in a Saudi Arabian intensive care unit. Crit Care. 2002;6(2):166‐174. 11983044 10.1186/cc1477 PMC111184

[19] Moon BH , Park SK , Jang DK , Jang KS , Kim JT , Han YM . Use of APACHE II and SAPS II to predict mortality for hemorrhagic and ischemic stroke patients. J Clin Neurosci. 2015;22(1):111‐115. 25172016 10.1016/j.jocn.2014.05.031

[20] Sakr Y , Krauss C , Amaral AB , et al. Comparison of the performance of SAPS II, SAPS 3, APACHE II, and their customized prognostic models in a surgical intensive care unit. Br J Anaesth. 2008;101(6):798‐803. 18845649 10.1093/bja/aen291

[21] Markgraf R , Deutschinoff G , Pientka L , Scholten T . Comparison of acute physiology and chronic health evaluations II and III and simplified acute physiology score II: a prospective cohort study evaluating these methods to predict outcome in a German interdisciplinary intensive care unit. Crit Care Med. 2000;28(1):26‐33. 10667495 10.1097/00003246-200001000-00005

[22] Xue WY , Xu YC , Wu YW , Yang M . Observation of elevated fasting blood glucose and functional outcome after ischemic stroke in patients with and without diabetes. Oncotarget. 2017;8(40):67980‐67989. 28978089 10.18632/oncotarget.19074 PMC5620229

[23] Sung JY , Chen CI , Hsieh YC , et al. Comparison of admission random glucose, fasting glucose, and glycated hemoglobin in predicting the neurological outcome of acute ischemic stroke: a retrospective study. PeerJ. 2017;5:e2948. 28168113 10.7717/peerj.2948 PMC5292024

[24] Grelli KN , Gindville MC , Walker CH , Jordan LC . Association of blood pressure, blood glucose, and temperature with neurological outcome after childhood stroke. JAMA Neurol. 2016;73(7):829‐835. 27214847 10.1001/jamaneurol.2016.0992

[25] Idicula TT , Waje‐Andreassen U , Brogger J , Naess H , Thomassen L . Serum albumin in ischemic stroke patients: the higher the better. The bergen stroke study . Cerebrovasc Dis. 2009;28(1):13‐17. 19420917 10.1159/000215938

[26] Babu MS , Kaul S , Dadheech S , Rajeshwar K , Jyothy A , Munshi A . Serum albumin levels in ischemic stroke and its subtypes: correlation with clinical outcome. Nutrition. 2013;29(6):872‐875. 23422540 10.1016/j.nut.2012.12.015

[27] Abubakar S , Sabir A , Ndakotsu M , Imam M , Tasiu M . Low admission serum albumin as prognostic determinant of 30‐day case fatality and adverse functional outcome following acute ischemic stroke. Pan Afr Med J. 2013;14:53. 23565300 10.11604/pamj.2013.14.53.1941 PMC3617615

[28] Bielewicz J , Kurzepa J , Czekajska‐Chehab E , et al. Worse neurological state during acute ischemic stroke is associated with a decrease in serum albumin levels. J Mol Neurosci. 2016;58(4):493‐496. 26757706 10.1007/s12031-015-0705-4 PMC4829619

[29] Kimura Y , Yamada M , Kakehi T , Itagaki A , Tanaka N , Muroh Y . Combination of low body mass index and low serum albumin level leads to poor functional recovery in stroke patients. J Stroke Cerebrovasc Dis. 2017;26(2):448‐453. 27856112 10.1016/j.jstrokecerebrovasdis.2016.10.008

[30] Sutter R , Grize L , Fuhr P , Ruegg S , Marsch S . Acute‐phase proteins and mortality in status epilepticus: a 5‐year observational cohort study. Crit Care Med. 2013;41(6):1526‐1533. 23507720 10.1097/CCM.0b013e318287f2ac

[31] Sutter R , Kaplan PW , Marsch S , Hammel EM , Ruegg S , Ziai WC . Early predictors of refractory status epilepticus: an international two‐center study. Eur J Neurol. 2015;22(1):79‐85. 25104078 10.1111/ene.12531

[32] Demertzi A , Soddu A , Laureys S . Consciousness supporting networks. Curr Opin Neurobiol. 2013;23(2):239‐244. 23273731 10.1016/j.conb.2012.12.003

[33] Trajman A , Luiz RR . McNemar chi2 test revisited: comparing sensitivity and specificity of diagnostic examinations. Scand J Clin Lab Invest. 2008;68(1):77‐80. 18224558 10.1080/00365510701666031

[34] Kosinski AS . A weighted generalized score statistic for comparison of predictive values of diagnostic tests. Stat Med. 2013;32(6):964‐977. 22912343 10.1002/sim.5587 PMC3756153

[35] Bland JM , Altman DG . Multiple significance tests: the Bonferroni method. BMJ. 1995;310(6973):170. 7833759 10.1136/bmj.310.6973.170 PMC2548561

[36] Matsuo K , Palmer JB . Anatomy and physiology of feeding and swallowing: normal and abnormal. Phys Med Rehabil Clin N Am. 2008;19(4):691‐707. 18940636 10.1016/j.pmr.2008.06.001 PMC2597750

[37] Macht M , White SD , Moss M . Swallowing dysfunction after critical illness. Chest. 2014;146(6):1681‐1689. 25451355 10.1378/chest.14-1133 PMC4251623

[38] Martino R , Foley N , Bhogal S , Diamant N , Speechley M , Teasell R . Dysphagia after stroke: incidence, diagnosis, and pulmonary complications. Stroke. 2005;36(12):2756‐2763. 16269630 10.1161/01.STR.0000190056.76543.eb

[39] Macht M , Wimbish T , Clark BJ , et al. Postextubation dysphagia is persistent and associated with poor outcomes in survivors of critical illness. Crit Care. 2011;15(5):R231. 21958475 10.1186/cc10472 PMC3334778

[40] Kornbluth J , Bhardwaj A . Evaluation of coma: a critical appraisal of popular scoring systems. Neurocrit Care. 2011;14(1):134‐143. 20652445 10.1007/s12028-010-9409-3

[41] Fischer M , Ruegg S , Czaplinski A , et al. Inter‐rater reliability of the full outline of unresponsiveness score and the Glasgow Coma Scale in critically ill patients: a prospective observational study. Crit Care. 2010;14(2):R64. 20398274 10.1186/cc8963 PMC2887186

[42] Iyer VN , Mandrekar JN , Danielson RD , Zubkov AY , Elmer JL , Wijdicks EF . Validity of the FOUR score coma scale in the medical intensive care unit. Mayo Clin Proc. 2009;84:694‐701. 19648386 10.4065/84.8.694 PMC2719522

[43] Su YY , Li X , Li SJ , et al. Predicting hospital mortality using APACHE II scores in neurocritically ill patients: a prospective study. J Neurol. 2009;256(9):1427‐1433. 19390767 10.1007/s00415-009-5129-z

[44] Rué M , Md AA , Álvarez M , Quintana S , Valero C . Performance of the Mortality Probability Models in assessing severity of illness during the first week in the intensive care unit. Crit Care Med. 2000;28(8):2819‐2824. 10966256 10.1097/00003246-200008000-00023

