# Supplementary material for: Development and validation of a new score for predicting functional outcome of neurocritically ill patients: The INCNS score
Source: CNS Neurosci Ther. 2019 Apr 10;26(1):21–9. doi: 10.1111/cns.13134 (PMC6930816; doi:10.1111/cns.13134)
Supplement: Supplementary file 1 [file CNS-26-21-s001.doc]

**Supplementary Materials for:**

**Development and validation of a new score for predicting functional outcome of neurocritically ill patients: the INCNS score**

Running title: A noval prognostic score for patients in NICU

Qiong Gao, MD^†^; Fang Yuan, MD, PhD^†^; Xi-ai Yang, MD; Ji-wen Zhu, MD; Lu Song, MD; Li-jie Bi, MD; Ze-yu Jiao, MD; Xiao-gang Kang, MD; Fang Yang, MD, PhD; Wen Jiang*, MD, PhD

Department of Neurology, Xijing Hospital, Fourth Military Medical University, Xi’an, 710032, PR China

^†^ Equal contributors

* Correspondence should be addressed to: Professor Wen Jiang, Department of Neurology, Xijing Hospital, Fourth Military Medical University, Xi'an 710032, PR China; E-mail: jiangwen@fmmu.edu.cn; Tel: +86-29-84771319; Fax: +86-29-3244986.

| **Content** |
| --- |
| **Table S1.** Patient characteristics at 24h in N-ICU |
| **Table S2.** Patient characteristics at 72h in N-ICU |
| **Table S3.** Etiology distribution |
| **Table S4.** Overall performances of the INCNS, APACHE II and SAPS II scoring systems |
| **Figure S1.** Comparisons of area under the receiver operating characteristic curve (AUC) for INCNS, Glasgow Coma Scale (GCS) and Full Outline of UnResponsiveness (FOUR) to discriminate the three-month functional outcome in neurocritically ill patients |
| **Figure S2.** Comparisons of area under receiver operating characteristic curve (AUC) for INCNS and National Institutes of Health Stroke Scale (NIHSS) to discriminate the three-month functional outcome in 502 stroke patients from our cohort of neurocritically ill patients |
|  |

| **Table S1.** **Patient characteristics at 24h in N-ICU** | | | | |
| --- | --- | --- | --- | --- |
| **Variable** | **Total**  **(n=941)** | **mRS:0-2 (n=384)** | **mRS:3-6 (n=557)** | **P** |
| Age, median (IQR), y | 52 (37, 64) | 45 (27, 56) | 58 (46, 70) | < 0.001 (Mann-Whitney *U*) |
| Gender, male, No. (%) | 564 (59.9) | 220 (57.3) | 344 (61.8) | 0.169 (χ^2^) |
| Hospital stay length, median (IQR), d | 15 (9, 23) | 15 (10, 22) | 15 (8, 24) | 0.529 (Mann-Whitney U) |
| N-ICU stay length, median (IQR), d | 10 (6, 16) | 9 (6, 14) | 11 (7, 18) | < 0.001 (Mann-Whitney U) |
| GCS, median (IQR), points | 12 (8, 15) | 14 (11, 15) | 10 (6, 14) | < 0.001 (Mann-Whitney U) |
| APACHE II, median (IQR), points | 9 (5, 15) | 6 (3, 10) | 12 (7, 17) | < 0.001 (Mann-Whitney U) |
| SAPS II, median (IQR), points | 23 (17, 32) | 18 (13, 23) | 27 (20, 37) | < 0.001 (Mann-Whitney U) |
| INCNS, median (IQR), points | 9 (6, 13) | 6 (4, 9) | 12 (8,16) | < 0.001 (Mann-Whitney U) |
| Inflammation |  |  |  |  |
| WBC (10^9^ / L), No. (%) |  |  |  | 0.806 (Fisher) |
| ≤ 2.8 | 3 (0.3) | 1 (0.3) | 2 (0.4) |  |
| 2.9 ~ 3.9 | 8 (0.9) | 3 (0.8) | 5 (0.9) |  |
| 4 ~ 10 | 497 (52.8) | 208 (54.2) | 289 (51.9) |  |
| 10.1 ~ 25.0 | 420 (44.6) | 165 (43.0) | 255 (45.8) |  |
| ≥ 25.1 | 13 (1.4) | 7 (1.8) | 6 (1.1) |  |
| Temperature (axillary,℃), No. (%) |  |  |  | 0.126 (Fisher) |
| ≤ 35.9 | 16 (1.7) | 4 (1.0) | 12 (2.2) |  |
| 36 ~ 38.4 | 829 (88.1) | 347 (90.4) | 482 (86.5) |  |
| 38.5 ~ 40 | 87 ( 9.2) | 32 (8.3) | 55 (9.9) |  |
| ≥ 40.1 | 9 (1.0) | 1 (0.3) | 8 (1.4) |  |
| Nutrition |  |  |  |  |
| Albumin (g / L), No. (%) |  |  |  | < 0.001 (Fisher) |
| ≤ 24.9 | 9 (1.0) | 3 (0.8) | 6 (1.1) |  |
| 25 ~ 34.9 | 211 (22.4) | 57 (14.8) | 154 (27.6) |  |
| ≥ 35 | 721 (76.6) | 324 (84.4) | 397 (71.3) |  |
| Consciousness |  |  |  |  |
| Arousal, No. (%) |  |  |  | < 0.001 (χ^2^) |
| Spontaneous eye opening | 521 (55.4) | 271 (70.6) | 250 (44.9) |  |
| Eye opening to verbal command | 171 (18.2) | 64 (16.7) | 107 (19.2) |  |
| Eye opening to pain | 113 (12.0) | 26 (6.8) | 87 (15.6) |  |
| None | 136 (14.5) | 23 (6.0) | 113 (20.3) |  |
| Awareness, No. (%) |  |  |  | < 0.001 (χ^2^) |
| Correct response to question or command | 325 (34.5) | 195 (50.8) | 130 (23.3) |  |
| Confused response to question or command | 230 (24.4) | 103 (26.8) | 127 (22.8) |  |
| Non-reflex movements | 310 (32.9) | 75 (19.5) | 235 (42.2) |  |
| None | 76 (8.1) | 11 (2.9) | 65 (11.7) |  |
| Neurological function |  |  |  |  |
| Pupillary light reflex, No. (%) |  |  |  | < 0.001 (χ^2^) |
| Bilateral sensitive | 766 (81.4) | 350 (91.1) | 416 (74.7) |  |
| Unilateral slow / absent | 67 (7.1) | 19 (4.9) | 48 (8.6) |  |
| Bilateral slow / absent | 108 (11.5) | 15 (3.9) | 93 (16.7) |  |
| Corneal reflex, No. (%) |  |  |  | < 0.001 (χ^2^) |
| Bilateral sensitive | 860 (91.4) | 374 (97.4) | 486 (87.3) |  |
| Unilateral slow / absent | 21 (2.2) | 5 (1.3) | 16 (2.9) |  |
| Bilateral slow / absent | 60 (6.4) | 5 (1.3) | 55 (9.9) |  |
| Verbal response, No. (%) |  |  |  | < 0.001 (χ^2^) |
| Accurate speech | 261 (27.7) | 182 (47.4) | 79 (14.2) |  |
| Confused / inappropriate speech | 283 (30.1) | 114 (29.7) | 169 (30.3) |  |
| Incomprehensible speech / none | 397 (42.2) | 88 (22.9) | 309 (55.5) |  |
| Motor response, No. (%) |  |  |  | < 0.001 (χ^2^) |
| Unilateral / bilateral muscle strength scores ≥ 4 or obeying to command | 291 (30.9) | 213 (55.5) | 78 (14.0) |  |
| Unilateral / bilateral muscle strength scores of 2-3 or localizing to /withdrawal from pain | 216 (23.0) | 90 (23.4) | 126 (22.6) |  |
| Unilateral muscle strength scores ≤  1or flexing / extending to pain | 322 (34.2) | 67 (17.4) | 255 (45.8) |  |
| Bilateral muscle strength scores ≤ 1 /  none | 112 (11.9) | 14 (3.6) | 98 (17.6) |  |
| Swallowing dysfunction, No. (%) |  |  |  | < 0.001 (χ^2^) |
| Water swallow test I - II | 450 (47.8) | 262 (68.2) | 188 (33.8) |  |
| Water swallow test III – IV /  unable to assess | 491 (52.2) | 122 (31.8) | 369 (66.2) |  |
| Spontaneous respiration, No. (%) |  |  |  | < 0.001 (Fisher) |
| Not intubated, ≤ 11 | 7 (0.7) | 3 (0.8) | 4 (0.7) |  |
| Not intubated, 12 ~ 24 | 566 (60.1) | 248 (64.6) | 318 (57.1) |  |
| Not intubated, ≥ 25 | 274 (29.1) | 116 (30.2) | 158 (28.4) |  |
| Breathes above ventilator rate | 77 (8.2) | 16 (4.2) | 61 (11.0) |  |
| Breathes at ventilator rate/ apnea | 17 (1.8) | 1 (0.3) | 16 (2.9) |  |
| Systemic function |  |  |  |  |
| Age (y), No. (%) |  |  |  | < 0.001 (χ^2^) |
| ≤ 44 | 317 (33.7) | 190 (49.5) | 127 (22.8) |  |
| 45 ~ 64 | 391 (41.6) | 149 (38.8) | 242 (43.4) |  |
| 65 ~ 74 | 137 (14.6) | 39 (10.2) | 98 (17.6) |  |
| ≥ 75 | 96 (10.2) | 6 (1.6) | 90 (16.2) |  |
| Heart rate, No. (%) |  |  |  | < 0.001 (Fisher) |
| ≤ 39 | 6 (0.6) | 2 (0.5) | 4 (0.7) |  |
| 40 ~ 59 | 187 (19.9) | 93 (24.2) | 94 (16.9) |  |
| 60 ~ 100 | 273 (29.0) | 127 (33.1) | 146 (26.2) |  |
| 101 ~149 | 452 (48.0) | 157 (40.9) | 295 (53.0) |  |
| ≥ 150 | 23 (2.4) | 5 (1.3) | 18 (3.2) |  |
| SBP (mmHg), No. (%) |  |  |  | < 0.001 (χ^2^) |
| ≤ 69 | 17 (1.8) | 0 (0) | 17 (3.1) |  |
| 70 ~ 89 | 67 (7.1) | 31 (8.1) | 36 (6.5) |  |
| 90 ~ 140 | 262 (27.8) | 137 (35.7) | 125 (22.4) |  |
| 141 ~ 199 | 558 (59.3) | 206 (53.6) | 352 (63.2) |  |
| ≥ 200 | 37 (3.9) | 10 (2.6) | 27 (4.8) |  |
| Serum glucose (mmol / L), No. (%) |  |  |  | 0.049 (Fisher) |
| ≤ 2.1 | 1 (0.1) | 0 (0) | 1 (0.2) |  |
| 2.2 ~ 3.8 | 17 (1.8) | 10 (2.6) | 7 (1.3) |  |
| 3.9 ~ 11.1 | 863 (91.7) | 358 (93.2) | 505 (90.7) |  |
| 11.2 ~ 19.3 | 52 (5.5) | 13 (3.4) | 39 (7.0) |  |
| ≥ 19.4 | 8 (0.9) | 3 (0.8) | 5 (0.9) |  |
| Serum sodium (mmol / L), No. (%) |  |  |  | 0.138 (Fisher) |
| ≤ 119 | 8 (0.9) | 5 (1.3) | 3 (0.5) |  |
| 120 ~ 129 | 61 (6.5) | 24 (6.3) | 37 (6.6) |  |
| 130 ~ 150 | 848 (90.1) | 350 (91.1) | 498 (89.4) |  |
| 151 ~ 159 | 13 (1.4) | 4 (1.0) | 9 (1.6) |  |
| ≥ 160 | 11 (1.2) | 1 (0.3) | 10 (1.8) |  |
| Serum potassium (mmol / L), No. (%) |  |  |  | 0.391 (Fisher) |
| ≤ 2.4 | 1 (0.1) | 1 (0.3) | 0 (0) |  |
| 2.5 ~ 3.4 | 129 (13.7) | 55 (14.3) | 74 (13.3) |  |
| 3.5 ~ 5.5 | 805 (85.5) | 327 (85.2) | 478 (85.8) |  |
| 5.6 ~ 6.9 | 6 (0.6) | 1 (0.3) | 5 (0.9) |  |
| ≥ 7.0 | 0 (0) | 0 (0) | 0 (0) |  |
| Serum creatinine (umol / L), No. (%) |  |  |  | 0.024 (Fisher) |
| ≤ 43 | 4 (0.4) | 1 (0.3) | 3 (0.5) |  |
| 44 ~ 132 | 881 (93.6) | 370 (96.4) | 511 (91.7) |  |
| 133 ~ 171 | 28 (3.0) | 5 (1.3) | 23 (4.1) |  |
| ≥ 172 | 28 (3.0) | 8 (2.1) | 20 (3.6) |  |
| Total bilirubin (umol / L), No. (%) |  |  |  | 0.055 (Fisher) |
| ≤ 34.1 | 883 (93.8) | 368 (95.8) | 515 (92.5) |  |
| 34.2 ~ 102.5 | 55 (5.8) | 16 (4.2) | 39 (7.0) |  |
| ≥ 102.6 | 3 (0.3) | 0 (0) | 3 (0.5) |  |

APACHE II, Acute Physiology and Chronic Health Evaluation II; GCS, Glasgow Coma Scale; IQR, interquartile range; mRS, Modified Rankin Scale; N-ICU, neurological intensive care unit; SAPS II, Simplified Acute Physiology Score II; SBP, systolic blood pressure; WBC, white blood cell.

| **Table S2.** Patient characteristics at 72h in N-ICU | | | | |
| --- | --- | --- | --- | --- |
| **Variable** | **Total**  **(n=941)** | **mRS:0-2 (n=383)** | **mRS:3-6 (n=558)** | **P** |
| GCS, median (IQR), points | 13 (8, 15) | 14 (11, 15) | 10 (6, 14) | < 0.001 (Mann-Whitney U) |
| APACHE II, median (IQR), points | 9 (5, 15) | 6 (4, 10) | 12 (7, 17) | < 0.001 (Mann-Whitney U) |
| SAPS II, median (IQR), points | 22 (15, 31) | 18 (13, 22) | 27 (20, 37) | < 0.001 (Mann-Whitney U) |
| INCNS, median (IQR), points | 9 (5, 14) | 5 (3, 8) | 12 (9, 16) | < 0.001 (Mann-Whitney U) |
| Inflammation |  |  |  |  |
| WBC (10^9^ / L), No. (%) |  |  |  | < 0.001 (Fisher) |
| ≤ 2.8 | 4 (0.4) | 3 (0.8) | 1 (0.2) |  |
| 2.9 ~ 3.9 | 11 (1.2) | 5 (1.3) | 6 (1.1) |  |
| 4 ~ 10 | 495 (52.6) | 233 (60.7) | 262 (47.0) |  |
| 10.1 ~ 25.0 | 415 (44.1) | 139 (36.2) | 276 (49.6) |  |
| ≥ 25.1 | 16 (1.7) | 4 (1.0) | 12 (2.2) |  |
| Temperature (axillary,℃), No. (%) |  |  |  | < 0.001 (Fisher) |
| ≤ 35.9 | 16 (1.7) | 3 0.8) | 13 (2.3) |  |
| 36 ~ 38.4 | 839 (89.2) | 365 (95.1) | 474 (85.1) |  |
| 38.5 ~ 40 | 80 ( 8.5) | 16 (4.2) | 64 (11.5) |  |
| ≥ 40.1 | 6 (0.6) | 0 (0) | 6 (1.1) |  |
| Nutrition |  |  |  |  |
| Albumin (g / L), No. (%) |  |  |  | < 0.001 (Fisher) |
| ≤ 24.9 | 15 (1.6) | 0 (0) | 15 (2.7) |  |
| 25 ~ 34.9 | 303 (32.2) | 77 (20.1) | 226 (40.6) |  |
| ≥ 35 | 623 (66.2) | 307 (79.9) | 316 (56.8) |  |
| Consciousness |  |  |  |  |
| Arousal, No. (%) |  |  |  | < 0.001 (χ^2^) |
| Spontaneous eye opening | 536 (57.0) | 283 (73.7) | 253 (45.4) |  |
| Eye opening to verbal command | 169 (18.0) | 64 (16.7) | 105 (18.9) |  |
| Eye opening to pain | 107 (11.4) | 21 (5.5) | 86 (15.4) |  |
| None | 129 (13.7) | 16 (4.2) | 113 (20.3) |  |
| Awareness, No. (%) |  |  |  | < 0.001 (χ^2^) |
| Correct response to question or command | 353 (37.5) | 218 (56.8) | 135 (24.2) |  |
| Confused response to question or command | 215 (22.8) | 93 (24.2) | 122 (21.9) |  |
| Non-reflex movements | 286 (30.4) | 65 (16.9) | 221 (39.7) |  |
| None | 87 (9.2) | 8 (2.1) | 79 (14.2) |  |
| Neurological function |  |  |  |  |
| Pupillary light reflex, No. (%) |  |  |  | < 0.001 (χ^2^) |
| Bilateral sensitive | 752 (79.9) | 352 (91.7) | 400 (71.8) |  |
| Unilateral slow / absent | 70 (7.4) | 19 (4.9) | 51 (9.2) |  |
| Bilateral slow / absent | 119 (12.6) | 13 (3.4) | 106 (19.0) |  |
| Corneal reflex, No. (%) |  |  |  | < 0.001 (χ^2^) |
| Bilateral sensitive | 846 (89.9) | 375 (97.7) | 471 (84.6) |  |
| Unilateral slow / absent | 20 (2.1) | 4 (1.0) | 16 (2.9) |  |
| Bilateral slow / absent | 75 (8.0) | 5 (1.3) | 70 (12.6) |  |
| Verbal response, No. (%) |  |  |  | < 0.001 (χ^2^) |
| Accurate speech | 270 (28.7) | 188 (49.0) | 82 (14.7) |  |
| Confused / inappropriate speech | 284 (30.2) | 116 (30.2) | 168 (30.2) |  |
| Incomprehensible speech / none | 387 (41.1) | 80 (20.8) | 307 (55.1) |  |
| Motor response, No. (%) |  |  |  | < 0.001 (χ^2^) |
| Unilateral / bilateral muscle strength scores ≥ 4 or obeying to command | 297 (31.6) | 223 (58.1) | 74 (13.3) |  |
| Unilateral / bilateral muscle strength scores of 2-3 or localizing to /withdrawal from pain | 139 (14.8) | 71 (18.5) | 68 (12.2) |  |
| Unilateral muscle strength scores ≤  1or flexing / extending to pain | 359 (38.2) | 75 (19.5) | 284 (51.0) |  |
| Bilateral muscle strength scores ≤ 1 /  none | 146 (15.5) | 15 (3.9) | 131 (23.5) |  |
| Swallowing dysfunction, No. (%) |  |  |  | < 0.001 (χ^2^) |
| Water swallow test I - II | 455 (48.4) | 267 (69.5) | 188 (33.8) |  |
| Water swallow test III – IV /  unable to assess | 486 (51.6) | 117 (30.5) | 369 (66.2) |  |
| Spontaneous respiration, No. (%) |  |  |  | < 0.001 (Fisher) |
| Not intubated, ≤ 11 | 11 (1.2) | 5 (1.3) | 6 (1.1) |  |
| Not intubated, 12 ~ 24 | 566 (60.1) | 256 (66.7) | 310 (55.7) |  |
| Not intubated, ≥ 25 | | 566 (60.1) | 258 (27.4) | 156 (28.0) | | --- | --- | --- | | | 566 (60.1) | 109 (28.4) | 156 (28.0) | | --- | --- | --- | | | 566 (60.1) | 149 (26.8) | 156 (28.0) | | --- | --- | --- | |  |
| Breathes above ventilator rate | 82 (8.7) | 14 (3.6) | 68 (12.2) |  |
| Breathes at ventilator rate/ apnea | 24 (2.6) | 0 (0) | 24 (4.3) |  |
| Systemic function |  |  |  |  |
| Heart rate, No. (%) |  |  |  | < 0.001 (χ^2^) |
| ≤ 39 | 16 (1.7) | 1 (0.3) | 15 (2.7) |  |
| 40 ~ 59 | 173 (18.4) | 89 (23.2) | 84 (15.1) |  |
| 60 ~ 100 | 296 (31.5) | 144 (37.5) | 152 (27.3) |  |
| 101 ~149 | 440 (46.8) | 148 (38.5) | 292 (52.4) |  |
| ≥ 150 | 16 (1.7) | 2 (0.5) | 14 (2.5) |  |
| SBP (mmHg), No. (%) |  |  |  | < 0.001 (χ^2^) |
| ≤ 69 | 28 (3.0) | 1 (0.3) | 27 (4.8) |  |
| 70 ~ 89 | 62 (6.6) | 22 (5.7) | 40 (7.2) |  |
| 90 ~ 140 | 313 (33.3) | 167 (43.5) | 146 (26.2) |  |
| 141 ~ 199 | 514 (54.6) | 189 (49.2) | 325 (58.3) |  |
| ≥ 200 | 24 (2.6) | 5 (1.3) | 19 (3.4) |  |
| Serum glucose (mmol / L), No. (%) |  |  |  | 0.001(χ^2^) |
| ≤ 2.1 | 0 (0) | 0 (0) | 0 (0) |  |
| 2.2 ~ 3.8 | 18 (1.9) | 9 (2.3) | 9 (1.6) |  |
| 3.9 ~ 11.1 | 842 (89.5) | 357 (93.0) | 485 (87.1) |  |
| 11.2 ~ 19.3 | 48 (5.1) | 14 (3.6) | 34 (6.1) |  |
| ≥ 19.4 | 33 (3.5) | 4 (1.0) | 29 (5.2) |  |
| Serum sodium (mmol / L), No. (%) |  |  |  | 0.001 (Fisher) |
| ≤ 119 | 3 (0.3) | 2 (0.5) | 1 (0.2) |  |
| 120 ~ 129 | 35 (3.7) | 10 (2.6) | 25 (4.5) |  |
| 130 ~ 150 | 880 (93.5) | 371 (96.6) | 509 (91.4) |  |
| 151 ~ 159 | 15 (1.6) | 1 (0.3) | 14 (2.5) |  |
| ≥ 160 | 8 (0.9) | 0 (0) | 8 (1.4) |  |
| Serum potassium (mmol / L), No. (%) |  |  |  | 0.124 (Fisher) |
| ≤ 2.4 | 3 (0.3) | 0 (0) | 3 (0.5) |  |
| 2.5 ~ 3.4 | 209 (22.2) | 76 (19.8) | 133 (23.9) |  |
| 3.5 ~ 5.5 | 720 (76.5) | 306 (79.7) | 414 (74.3) |  |
| 5.6 ~ 6.9 | 9 (1.0) | 2 (0.5) | 7 (1.3) |  |
| ≥ 7.0 | 0 (0) | 0 (0) | 0 (0) |  |
| Serum creatinine (umol / L), No. (%) |  |  |  | 0.026 (Fisher) |
| ≤ 43 | 4 (0.4) | 1 (0.3) | 3 (0.5) |  |
| 44 ~ 132 | 877 (93.2) | 369 (96.1) | 508 (91.2) |  |
| 133 ~ 171 | 23 (2.4) | 5 (1.3) | 18 (3.2) |  |
| ≥ 172 | 37 (3.9) | 9 (2.3) | 28 (5.0) |  |
| Total bilirubin (umol / L), No. (%) |  |  |  | 0.007 (Fisher) |
| ≤ 34.1 | 913 (97.0) | 380 (99.0) | 533 (95.7) |  |
| 34.2 ~ 102.5 | 26 (2.8) | 4 (1.0) | 22 (3.9) |  |
| ≥ 102.6 | 2 (0.2) | 0 (0) | 2 (0.4) |  |

APACHE II, Acute Physiology and Chronic Health Evaluation II; GCS, Glasgow Coma Scale; IQR, interquartile range; mRS, Modified Rankin Scale; N-ICU, neurological intensive care unit; SAPS II, Simplified Acute Physiology Score II; SBP, systolic blood pressure; WBC, white blood cell.

| **Table S3.** Etiology distribution | | | |
| --- | --- | --- | --- |
| **Aetiology** | **Total**  **No. (%)** | **mRS (0-2)**  **No. (%)** | **mRS (3-6)**  **No. (%)** |
| Cerebral infarction | 350 (37.2) | 120 (31.3) | 230 (41.2) |
| Intracerebral hemorrhage | 152 (16.2) | 56 (14.6) | 96 (17.2) |
| Neurologic inflammation | 214 (22.7) | 127 (33.2) | 87 (15.6) |
| Status epilepticus | 31 (3.3) | 17 (4.4) | 14 (2.5) |
| Guillain-barre syndrome | 34 (3.6) | 15 (3.9) | 19 (3.4) |
| Traumatic brain injury | 19 (2.0) | 7 (1.8) | 12 (2.2) |
| Poisoning | 12 (2.2) | 15 (3.9) | 22 (3.9) |
| Hypoxic ischemic encephalopathy | 25 (2.7) | 3 (0.8) | 22 (3.9) |
| Other neurologic diseases | 79 (8.4) | 23 (6.0) | 56 (10.0) |

| **Table S4.** Overall performances of the INCNS, APACHE II and SAPS II scoring systems | | | | | | | | |
| --- | --- | --- | --- | --- | --- | --- | --- | --- |
| **Score** | **AUC (95% CI)** | **Cut-off values** | **Se** | **Sp** | **PPV** | **NPV** | **CC** | **Accuracy** |
| INCNS 24h | 0.788  (0.759 – 0.817) | 9 | 73.8 | 71.9 | 79.2 | 65.4 | 73.0 | 72.9 |
| 72h | 0.828  (0.802 – 0.854) | 9 | 75.0 | 76.0 | 82.0 | 67.7 | 75.5 | 75.5 |
| APACHE II 24h | 0.749  (0.718 – 0.780) | 11 | 57.5 | 80.7 | 81.2 | 56.7 | 67.0 | 69.1 |
| 72h | 0.756  (0.726 – 0.787) | 11 | 58.4 | 81.8 | 82.2 | 57.4 | 67.8 | 70.1 |
| SAPS II 24h | 0.757  (0.727 – 0.788) | 23 | 69.1 | 72.4 | 78.4 | 61.8 | 70.5 | 70.8 |
| 72h | 0.770  (0.740 – 0.800) | 23 | 67.0 | 75.3 | 79.7 | 61.1 | 70.4 | 71.2 |

AUC, area under the curve; CC, correctly classified; NPV, negative predictive value; PPV, positive predictive value; Se, sensitivity; Sp, specificity.


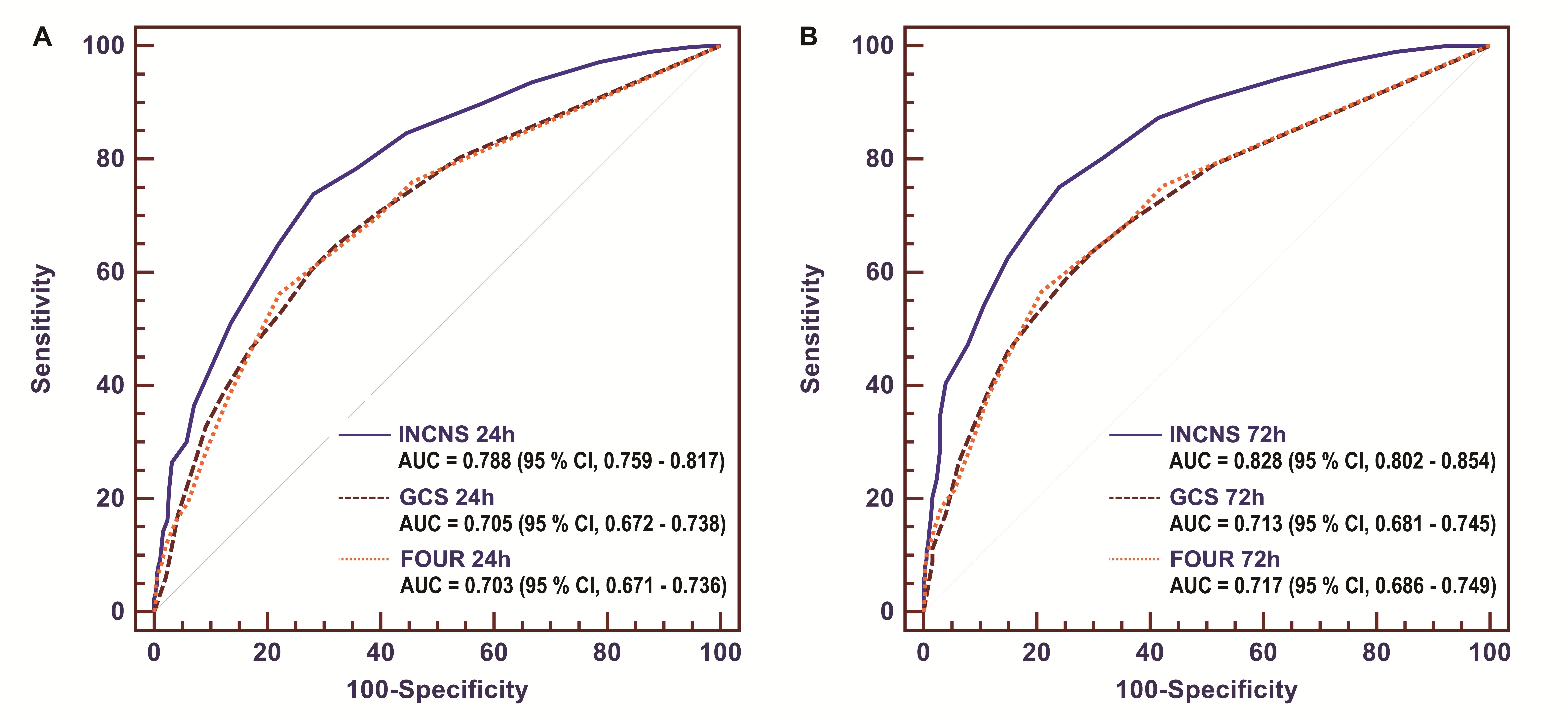


**Figure S1.** Comparisons of area under the receiver operating characteristic curve (AUC) for INCNS, Glasgow Coma Scale (GCS) and Full Outline of UnResponsiveness (FOUR) to discriminate the three-month functional outcome in neurocritically ill patients. A) ROC at 24 hours: the p value for the comparison of AUC between INCNS and GCS is less than 0.0001, between INCNS and FOUR is also less than 0.0001. B) ROC at 72 hours: the p value for the comparison of AUC between INCNS and GCS is less than 0.0001, between INCNS and FOUR is also less than 0.0001. Level of significance corrected for multiple testing p < 0.0167.


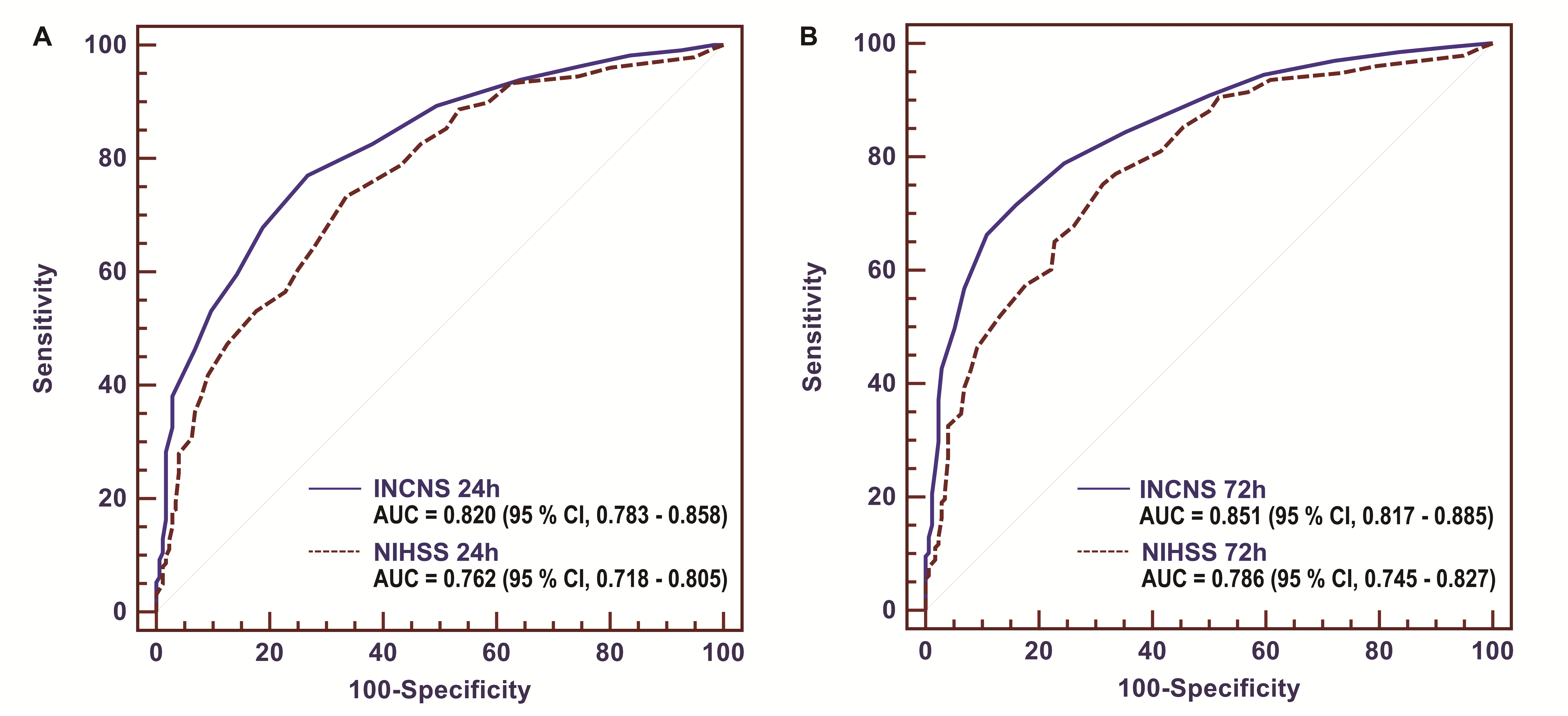


**Figure S2.** Comparisons of area under receiver operating characteristic curve (AUC) for INCNS and National Institutes of Health Stroke Scale (NIHSS) to discriminate the three-month functional outcome in 502 stroke patients from our cohort of neurocritically ill patients. A) ROC at 24 hours: the p value for the comparison of AUC between INCNS and NIHSS is 0.0015. B) ROC at 72 hours: the p value for the comparison of AUC between INCNS and NIHSS is 0.0003. Level of significance corrected for multiple testing p < 0.05.
